# Pharmaceutical solutions implemented to improve mental health care pathways: a systematic realist review protocol

**DOI:** 10.1136/bmjopen-2025-110428

**Published:** 2025-09-30

**Authors:** Matthieu Lebrat, Florence Carrouel, Nicolas Franck, Claude Dussart

**Affiliations:** 1 Pharmacie et Stérilisation Centrales, Hospices Civils de Lyon, F-69000, Lyon, France; 2 Laboratory ‘Health Systemic Process’ (P2S), UR 4129, Claude Bernard University, University of Lyon, Villeurbanne, France; 3 Claude Bernard University, University of Lyon, Villeurbanne, France; 4 Centre Ressource de Réhabilitation psychosociale, Pôle Centre Rive Gauche, Le Vinatier Hospital, Bron, France; 5 UMR 5229 CNRS, Le Vinatier Hospital, Bron, France

**Keywords:** MENTAL HEALTH, Pharmacists, Health Services, Primary Health Care, Hospital to Home Transition, Systematic Review

## Abstract

**Abstract:**

**Introduction:**

Care pathways are crucial for patients with mental health disorders and should be designed to support integrated rehabilitation while reducing the burden of these disorders. The contemporary shift toward an outpatient follow-up model of care presents an opportunity to improve mental health care beyond the stagnation in advancements in pharmacological treatments. Various pharmacist-led interventions exist and can serve as levers to address ongoing challenges in mental health care pathways: they could help manage difficult transitions, ensure continuity between inpatient and outpatient care, and reduce high rehospitalisation rates. However, the contexts in which these solutions benefit patients and improve care outcomes remain unclear. Thus, the primary objective of this study will be to identify how pharmaceutical solutions contribute to improving mental health care pathways, what works, for whom and in what context. The secondary objective will be to identify the key outcomes currently used to evaluate the impact of pharmaceutical solutions on care pathways.

**Methods and analysis:**

A systematic realist review will be conducted, following 5 iterative steps to synthesise heterogeneous evidence: (1) Scope definition with a general review of the literature and experts’ discussions, (2) Initial programme theory development based on the preliminary searches, (3) Systematic review for evidence, to refine and test initial programme theory across PubMed, Embase and Web Of Science, (4) Data extraction, including context-mechanism-outcome configurations, and evidence appraisal and (5) Data analysis, synthesis and refined programme theory construction with the realist logic. This process will involve consensus among expert researchers, incorporating insights from individuals with lived experience.

The final programme theory modelling will result in a new framework for pharmaceutical solutions applied in diverse mental health contexts. The findings of this systematic realist review could serve as a guide for implementing pharmaceutical solutions across healthcare settings, ensuring that interventions are evidence-based, contextually relevant and grounded in real-world needs.

**Ethics and dissemination:**

As this realist review will collect previously published data and will not involve human or animal participants, no ethical approval is required. Since this manuscript is a review protocol, no datasets were generated or analysed. All data extraction forms will be made available as part of the publication of the realist review.

**PROSPERO registration number:**

Systematic review registration PROSPERO 2025 CRD420251011954.

Dates of the study: September 2025 to September 2026.

**STRENGTHS AND LIMITATIONS OF THIS STUDY**

- The realist systematic review design provides a robust tool for synthesising heterogeneous evidence on pharmaceutical solutions across diverse mental health contexts.
- Protocol development follows established guidelines for both realist and systematic reviews.
- Holistic and integrative approach, incorporating multiple inputs from experts and individuals with lived experience to ensure alignment with real-world needs and priorities.
- Anticipated limitations in the generalisability of the refined programme theory and synthesis due to disparities in healthcare systems.

## Introduction

 As described in the 11th Revision of the International Classification of Diseases, mental health disorders encompass a range of disorders such as depression, bipolar disorders, anxiety disorders and schizophrenia.^1^ Characterised by clinically significant disturbances in an individual’s cognition, emotional regulation or behaviour, they represent a major global public health challenge, affecting one in eight people worldwide.^2^ These disorders are among the leading causes of disability in both high-income and low-income to middle-income countries, contributing significantly to morbidity, mortality and economic loss.^3^ According to the global burden of disease study, 7 of the top 25 leading causes of global years lived with disability (YLDs) are mental health disorders and accounted for 17.2% of total YLDs worldwide in 2021.^4^ This situation has worsened lately due to the COVID-19 pandemic, which has exacerbated both the prevalence and severity of symptoms for many individuals, leading to a rise in mental healthcare utilisation and psychiatric medication prescriptions.^5^

In response to this public health burden, optimising care pathways for patients with mental health disorders has become critical.^6^ An effective and consistent care pathway is essential not only to ensure timely and appropriate access to care but also to improve the quality of care throughout the patient’s health pathway.^7^ People with mental health conditions typically move through a range of care pathways that vary in intensity and duration depending on clinical need. After diagnosis, usually by primary care providers, patients may be referred to one or more of the following settings: acute inpatient care for severe episodes (such as the emergency department), standard longer-term inpatient care for a global therapeutic strategy decision or adaptation, and outpatient or community-based services for ongoing management and rehabilitation.^8^,^10^ Patients may move between these settings to achieve therapeutic goals, with long-term follow-up often provided by multidisciplinary teams in primary or community care, focusing on patient-centred rehabilitation and relapse prevention.

Unfortunately, mental health care pathways are often fraught with significant challenges, including gaps in care continuity,^10 11^ persistent iatrogenic risks^12 13^ and lack of resources and multidisciplinary collaboration among health professionals.^14^ As a result, many patients encounter barriers that prevent them from accessing proper treatment or adhering to treatment regimens, leading to frequent readmissions and inefficient rehabilitation.^9 15^ Improving mental health care pathways is therefore a crucial lever for reducing access disparities, enhancing clinical outcomes and supporting successful social reintegration for patients.^4 6^

Pharmacists are among the most accessible healthcare providers and are key stakeholders in healthcare systems, ensuring access to care through their extensive community-based network.^16^ They work across a range of contexts, including hospitals and privately run commercial/community pharmacies, where their access to patients and resources may differ, influencing the scope of care they can provide. With an average of 58 to 123 pharmacists per 100 000 inhabitants across most European countries in 2022,^17^ they contribute to prevention, follow-up and treatment, making pharmaceutical solutions particularly valuable in mental health care pathways.^18^ Pharmacists frequently encounter individuals with mental health concerns, sometimes even prior to diagnosis. For example, community pharmacists have reported seeing individuals with symptoms suggestive of anxiety or depression before a formal diagnosis.^19^

Specifically, pharmacists provide pharmaceutical care, originally defined in the 1990s as ‘the direct, responsible provision of medication-related care for the purpose of achieving definite outcomes’.^20^ Over time, the concept has broadened to encompass a wider range of pharmacist-led services that improve multiple health outcomes, not solely those related to drug therapy.^21 22^ Thus, pharmaceutical care represents a shift from merely dispensing medications to proactively managing therapy within a patient-centred approach aimed at optimising health outcomes. Pharmaceutical professionals themselves have been rethinking their role within care pathways, and the literature supports evidence of pharmacists’ desire to better bridge the multiple unmet needs in mental healthcare.^18 23 24^

The scope of this work has therefore been limited to studying the contribution of pharmaceutical care through specifically designed solutions intended to enhance mental health care pathways.

**Figure 1:**
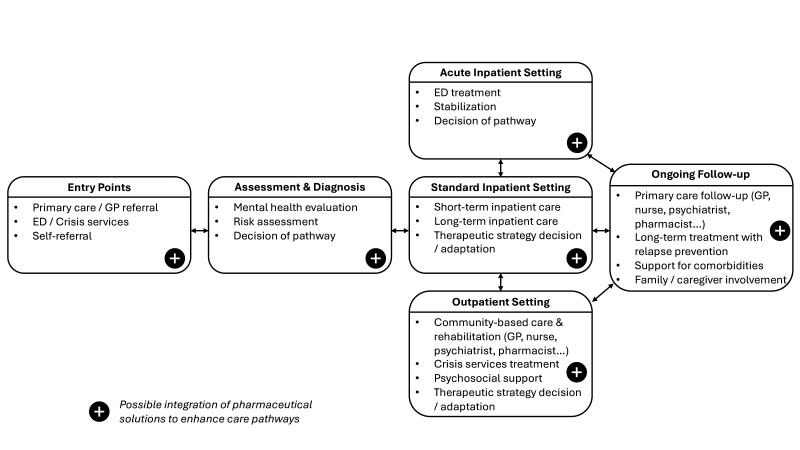
Simplified mental healthcare pathway showing entry points, care settings and transitions. ED, Emergency Department; GP, General Practitioner.

In this work, pharmaceutical solutions were defined as specific pharmacist-led interventions with a clearly delimited scope of action, which can be considered a sub-component of pharmaceutical care. These solutions, developed by pharmacists, can include: targeted pharmaceutical consultations (pharmacist-led meetings with patients on a specific issue, eg, antidepressant initiation), discharge workshops (inpatient workshops led by pharmacists in which patients preparing for discharge receive both individualised and general advice on their medications to support continuity of care), and outpatient pharmaceutical follow-ups (specific follow-ups by community pharmacists at each medication refill). Such solutions are key elements in improving care pathways, serving as levers to enhance both healthcare quality and accessibility. Indeed, pharmaceutical solutions are particularly valuable in mental health, where medication regimens are complex, side effects are significant and adherence is often suboptimal. They are closely linked to significant aspects of care pathways, such as rehospitalisation, therapeutic outcomes and care transitions. However, these solutions are often complex, consisting of multiple sub-interventions implemented across diverse settings, which complicates the analysis of the causal mechanisms underlying their effectiveness.^25^,^27^ Figure 1 provides an overview of a simplified care pathway through the mental health system, clarifying the contexts in which pharmaceutical solutions may play a role.

In practice, for pharmaceutical solutions to be fully effective, they must be integrated into care pathways, considering individual patients’ characteristics, their specific needs and available healthcare resources. Organisational leaders and health professionals require high-quality information on pharmaceutical solutions and their causal relationship with expected mental health care pathway outcomes to make informed decisions about their design and implementation. A realist review offers a framework for this analysis by identifying a programme theory, defined as context-mechanism-outcome (CMO) relationships.^28^,^30^

This approach will support a clearer understanding of how mental health outcomes can be improved by optimising care pathways, while also providing a structure to interpret our review findings. Within this broader objective, we will focus on the role of pharmaceutical solutions as one component of system-wide strategies in mental health. Accordingly, a systematic review will be conducted to identify pharmaceutical solutions that have been implemented to strengthen care pathways. The primary objective of this study is to determine how such pharmaceutical solutions contribute to improving outcomes for people with mental health conditions, specifically, what works, for whom and in what context. The secondary objective will be to identify the key outcomes currently used to evaluate the impact of pharmaceutical solutions on care pathways.

## Materials and methods

### Study design—systematic realist review

Pharmaceutical solutions are inherently complex, as they often involve multiple subprocesses and engage a range of stakeholders.^14 25 27 31^ Moreover, they are delivered by humans, each bringing unique approaches and variations in care delivery, which adds further complexity to their implementation and effectiveness.^32^ To develop a clear understanding of how pharmaceutical solutions work, and how they can be most effectively implemented within a system-wide approach, it is necessary to assess the relationship between the context in which the interventions are applied, the mechanisms by which they work and the outcomes which are produced. A realist review is specifically designed for this purpose, providing a valuable tool for synthesising heterogeneous and multi-component evidence to analyse complex pharmaceutical solutions across diverse contexts.^30 33^ Although systems-thinking approaches are relevant for higher-level policy design, they do not provide the same level of explanatory detail regarding why an intervention works for some groups and not others. The realist logic of inquiry has been successfully applied in mental health research (eg, Howe *et al*, BMJ Qual Saf 2023^13^) and is therefore appropriate for the present study. Importantly, the insights generated from this realist review could subsequently inform and complement systems-thinking approaches, ensuring that both methodological perspectives contribute to a more integrated understanding of mental health care pathways.

The realist approach consists of building, testing and refining theories regarding complex interventions and investigating how interactions are made with specific contexts to produce outcomes.^34^ It helps to go beyond individual evaluations of the interventions and explore the underlying entities, processes or structures which operate in particular situations.^29^ Indeed, randomised controlled trials (RCTs) or meta-analyses label the outcome of an intervention as effective, not effective or inconclusive, without providing information on the elements that could have led to the intervention’s success or failure.^30^ This dearth of information can be particularly challenging when dealing with complex interventions, such as pharmaceutical solutions, which integrate different variables implemented at multiple levels within the healthcare system.^35^ Health professionals and policymakers can thus lack information regarding the evidence-based implementation of pharmaceutical solutions.

**Figure 2:**
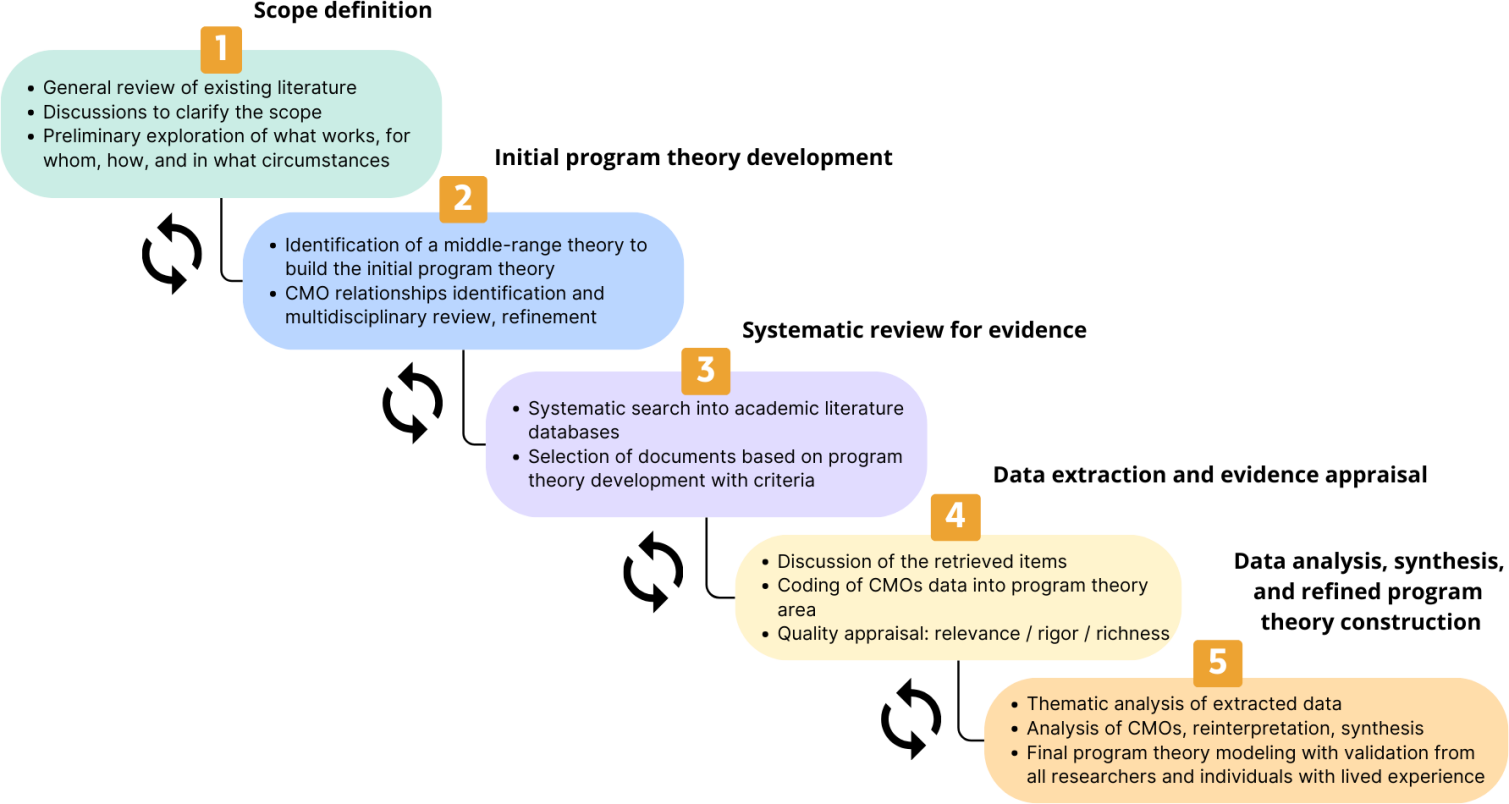
Study protocol flow chart. CMO, Context-Mechanism-Outcome.

We designed this systematic realist review protocol in collaboration with researchers specialising in mental health, pharmaceutical sciences, healthcare pathways and literature review. Along with evidence from published studies, input from stakeholders will be considered throughout this study to achieve its objectives. Additionally, input from individuals with lived experience will be incorporated at every stage of the study, from its design to the interpretation of the findings, through collaboration with a patient association. The developed protocol was based on and adapted from the following 5 iterative steps described by Pawson and colleagues, which are commonly found in similar protocols:^28 36 37^ (1) Scope definition, (2) Initial programme theory development, (3) Systematic review for evidence, (4) Data extraction and evidence appraisal and (5) Data analysis, synthesis and refined programme theory construction. Figure 2 summarises the steps outlined in our systematic realist review.

This realist review protocol was designed in accordance with the realist and meta-narrative evidence synthesis: evolving standards.^29 38^ Preferred Reporting Items for Systematic Reviews and Meta-Analyses Protocol (PRISMA-P)^39 40^ recommendations were followed to report the search strategy and study selection process (online supplemental table S1 PRISMA-P Checklist). The present protocol was registered in PROSPERO (CRD420251011954: 03/21/2025).

#### Step 1: scope definition

This step aims to gather key insights into the scope of existing pharmaceutical solutions within mental healthcare pathways and provide us with a starting point for future programme theory development. A preliminary general review will be conducted. The search terms ‘pharmacy’, ‘pharmaceutical’, ‘psychiatry’, ‘mental health’, ‘clinical pharmacy’, ‘pharmaceutical care’, (and their synonyms) with proper Boolean operators will be used in PubMed, Web of Science and Embase. Through literature examination, backward citation searching will be done. National (published in French) as well as international documents and guidelines (published in English) on pharmaceutical solutions in the context of mental health care will also be considered when data reflects enough trustworthiness and credibility.^41^ Discussions among the expert researchers and their academic research unit, specialised in healthcare pathways, will also be conducted to clarify the scope.

This preliminary search will help with the building of initial programme theories, which is the first step in designing a framework to further synthesise evidence.^28^ An exploration will be made of ‘what works, for whom, how and in what circumstances’.^28 29^ Thus, this first step will help to investigate the diversity of pharmaceutical solutions improving care pathways for patients with a psychiatric disorder. It will also allow us to examine the mechanisms through which they contribute to successful outcomes for the different stakeholders considered, and the conditions for it. The different types of outcomes related to care pathways will also be studied.

#### Step 2: initial programme theory development

Based on data obtained in step 1 and the initial literature scoping, the authors will determine suitable programme theories. These are conceptual frameworks or diagrams that outline the components of a programme or intervention and explain how they are intended to work.^42^ To do so, using a middle-range theory can be a valuable theoretical framework to build an initial programme theory.^29 30^ A middle-range theory is ‘a theory that is specific enough to generate hypotheses (for example in the form of propositions) to be tested in a particular case, or to help explain findings in a particular case, but general enough to apply across a number of cases or a number of domains’.^42^ It will help investigate how, for whom and under which contexts pharmaceutical solutions improve the care pathways for patients with a psychiatric disorder. The conceptual framework elaborated by Scahill *et al*^43^ adapted for pharmacy practice and mental health, will serve as a middle-range theory in our work. However, given the complexity of these solutions, a unique middle-range theory might not be sufficient to support the theory-building process within our realist systematic review.^44^ Thus, after step 1, other middle-range theories and theoretical statements considered valuable might be used and tested against the literature findings to support the present study.

Discussions among researchers will lead to the selection of the best theories reflecting their explanatory strength regarding improvement of mental health care pathways with pharmaceutical solutions. After this first selection, CMO relationships will be established and further refined: C (Contexts), the situations, conditions or environments in which pharmaceutical solutions are effective and trigger underlying mechanisms; M (Mechanisms), how pharmaceutical solutions work and link C to O; O (Outcomes), the different patterns of outcomes on care pathways associated with pharmaceutical solutions.

All candidate programme theories and CMO configurations will be reviewed by all authors and discussed until consensus is reached. As in step 1, discussions with the expert researchers and their affiliated academic research unit will also be undertaken to gather their feedback. A patient’s association representative comments will also be considered for initial programme theory development. Indeed, to take their opinion into account is central to prioritise and understanding emerging theory areas and CMOs.^45^ Inputs regarding (1) The expected benefits of pharmaceutical solutions, (2) Their need from pharmaceutical care and (3) How solutions should best be implemented will be collected from the patient’s association to guide preliminary theory development.

Key generic questions, along with initial programme theory, will be generated at this step to be used for the selection of evidence in the next steps and to better understand CMO configurations.

#### Step 3: systematic review for evidence

A systematic review method of the literature will be undertaken following step 2 to refine and test the initial programme theory.

#### Search strategy

PubMed, Embase and Web of Science databases will be searched from 2015 up to the search date. Search terms used for this step will be used to systematically collect references focused on pharmaceutical solutions in the context of mental health. Multiple combinations of the following words, with their Medical Subject Headings (MeSH) terms and free text terms will be used with Boolean operators for inclusion: (1) Pharmaceutical solutions including any pharmaceutical care action (‘pharmacist-led intervention’ and synonyms) targeting patients with, (2) Mental disorders (‘mental disorder’ and synonyms) and (3) Care pathways (‘care pathway’ and synonyms). Pharmacological interventions, including the effects of a drug, will not be considered as a pharmaceutical solution in this work. Considering the combinations of our search terms, this review will cover the full mental health care pathway, but the analysis will be structured around pharmaceutical solutions within these pathways. This approach will ensure a comprehensive yet bounded scope.

Search terms for this purposive search method and their specificities for each database are described in (online supplemental file S2 Search terms) and have been validated by a specialised librarian.

Additional articles will be identified through reference scanning of the collected articles, as well as recommendations from expert researchers consulted for this work, if applicable, during the study. Grey literature will not be considered, given the large number of references expected from the international scope of this study.

All identified records will be exported to Rayyan, a systematic review management software, for data selection and duplicate removal. The remaining citations and their metadata will then be imported into Zotero, a citation management software, for the next steps of the study.

### Inclusion and exclusion criteria

A preliminary consideration on inclusion and exclusion criteria has been conducted to select studies, as outlined in table 1. No selection will be made a priori on the publication type to stay aligned with the realist review philosophy.^28 29^ All peer-reviewed documents related to pharmaceutical solutions aimed at improving mental health care pathways that provide data to inform programme theory development and refinement will be considered, regardless of their methodology. This process will enable the analysis and synthesis of a broad range of study methodologies (including RCTs, qualitative studies or case studies).

**Table 1:** Preliminary inclusion and exclusion criteria

Inclusion criteria	Exclusion criteria
Documents in French or English Qualitative/quantitative/mixed-method design Focus on a pharmaceutical intervention Solution carried out for patients with a mental health disorder Outcomes on care pathways reported	Documents in languages other than French or English Solution not involving a pharmaceutical professional Solution not designed for patients with a mental health disorder Focus on a pharmacological intervention No reported outcomes on care pathways Published before 2015

After steps 1 and 2, a final list of inclusion and exclusion criteria will be established, and the criteria in table 1 may be updated accordingly.

Two stages will be undertaken to screen and select the documents by two independent researchers (ML and FC). First, ML will screen the documents based on titles and abstracts and, if considered relevant, will then review the full text against the inclusion and exclusion criteria. For documents without an abstract, a whole document reading will be conducted directly. Second, to reduce bias, FC will independently screen a random sample of 10% of the selected studies (adapted from techniques in references^37 46 47^) and confirm their eligibility and relevance for inclusion. If consensus cannot be reached for a document, a discussion will be held, and a third researcher (CD) will be consulted to obtain it. Documents in French-language will be screened, extracted and analysed in the same manner as English-language articles. Data from French articles will be translated into English during extraction to ensure consistency and facilitate synthesis.

#### Step 4: data extraction and evidence appraisal

All relevant extracted data will be exported into an Excel form by one author (ML), made in accordance with the realist review reference methodology.^29 48^ Another author (FC) will validate data extraction and review a 10% random subsample of documents for consistency. The extracted data will help to answer the research question of this work:^29^ how pharmaceutical solutions are effective, what works, for whom and in what context. The following data will be extracted for each included document: general information (eg, title, authors, publication date, location), study characteristics (eg, study type, aim, design, participants, diagnostic category, setting, interventions, outcomes, limitations and conclusions), intervention(s) characteristics (eg, type, levels of involvement of pharmaceutical staff, health system component) and CMO configurations (key elements).

The retrieved items will be discussed and refined within the research team as necessary during the study. Extracted data regarding CMO configurations will be coded into the most relevant programme theory area.

#### Quality appraisal

Simultaneously with data extraction, a critical quality appraisal is needed to ensure the coherence, plausibility and appropriateness of the processes used to inform data extraction judgments.^29^ In line with the realist philosophy, the quality assessment will not rely on the hierarchy of evidence or methodology, but on its applicability to inform the theory and the understandings of pharmaceutical solutions in the mental health area to improve healthcare pathways. Two regular criteria will help with this stage: relevance (whether a document can contribute to theory building and/or testing) and rigour (whether the method used to generate that particular piece of data is credible and trustworthy).^29^ A third criterion, ‘richness’, will also be added, as it has recently been discussed as a new criterion of interest for reporting evidence quality appraisal.^41^ Richness will be considered high when the resource meaningfully contributes to theory development and/or testing.^41^ Thus, a process including an assessment of relevance, richness and rigour in the evidence appraisal step will be conducted (scored as ‘high’ or ‘low’ for each criterion), adapted from the proposed considerations of Dada *et al*.^41^

#### Step 5: data analysis, synthesis and refined programme theory construction

This step will involve an iterative analysis of the collected data to examine different CMO configurations related to pharmaceutical solutions for improving mental health care pathways and refining the programme theory. The focus will be on identifying and aligning evidence from previous steps to demonstrate how specific mechanisms generate particular outcomes and to analyse which contextual factors are essential.^42^ Throughout this part, a realist philosophical ‘lens’ will be applied to the data using realist concepts, focusing on a generative explanation of causation within CMO configurations. We will use an approach that combines realist standards^29 38^ with adapted analysis methods, such as those described by Moons *et al*^36^ and Abayneh *et al*,^37^ as they align with our study design:

1. *Pooling of extracted data from the included documents in a tabulated table*. This will provide a first summary of the research question evidence. Data from step 4 will be organised based on relevance, richness and rigour.
2. *Thematic analysis*. Based on the elements of the data challenging the initial programme theory, a thematic analysis will be undertaken. All documents will be integrally analysed in order to identify each CMO configuration and reinterpret them. Documents with the highest quality criteria will be analysed independently by 2 authors (ML and FC). Any disagreements will be discussed with a third author (CD), and meetings will be held with the authors to standardise their analytical approach and guarantee trustworthiness. The remaining documents will be analysed by the first author, with regular input from the second author.
3. *Themes, organisation and chains of inference formulation*. Together with the entire research team, the previously identified themes will be validated based on their depth in informing the programme theory, and chains of inferences will be constructed across the different documents. We anticipate that new relationships within CMOs may emerge at this stage, which will be discussed and reflected on.
4. *Pattern of CMO identification and programme theory refinement*. A synthesis on the different CMOs will be conducted through discussions with all expert researchers, enabling a final programme theory refinement. Feedback from a patient association will also be considered to gain insights from individuals with lived experience. The CMOs will be tested and challenged with the data from the included documents to assess their explanatory strength. Thus, final programme theory modelling regarding pharmaceutical solutions aimed at improving mental health care pathways will be made possible within a newly established framework.

### Patient and public involvement

Patient involvement is embedded throughout the protocol via collaboration with a patient association, ensuring that the perspectives of individuals with lived experience inform the study. Their priorities, experiences and preferences will guide the development and refinement of the programme theory, with particular attention to shaping the emerging context–mechanism–outcome configurations.

The present protocol has been presented to the members of the author’s academic research unit, ‘Health Systemic Process’ (P2S), UR 4129, University Claude Bernard Lyon 1, University of Lyon, Lyon, France, for comments, one of whom is a patient partner.

## Discussion

This systematic realist review protocol aims to address a critical gap in the literature by identifying how pharmaceutical solutions improve mental health care pathways, while also exploring what works, for whom, and in what contexts they function. Adopting a realist, theory-driven and iterative 5-step approach, this study seeks to synthesise heterogeneous evidence and develop a refined programme theory that accounts for the complexity of pharmaceutical interventions in mental health settings and their impact on care pathways. Yet, no academic data synthesising the pharmaceutical solutions used internationally in mental health is available, or has a comprehensive realist review been conducted in this field. This knowledge gap underscores the need for a study on this research question that would allow to prioritise pragmatic pharmaceutical interventions to develop and implement in different contexts of the healthcare pathways in mental health.

Pharmaceutical solutions, including a wide range of possible interventions, hold the potential to optimise care pathways by enhancing care continuity, reducing rehospitalisation rates and addressing transitions between inpatient and outpatient care. Despite the recognised role of pharmacists in supporting mental health care, the literature on pharmacist-led interventions is scarce, primarily focused on inpatient settings rather than covering the full spectrum of care pathways.^31 43^ Pharmacists, as part of an evolving healthcare system, recognise their potential to address public health needs and are eager to take on high-value roles in mental health care beyond the stand-alone provision of medication and related advice.^23 24^ In order to support this change, data must be obtained on (1) Potential new interventions that pharmacists can take part in to enhance mental health care, (2) How these activities can be successfully integrated within a broader, system-wide approach and (3) Which outcomes should be used to evaluate their impact on care pathways.

The realist systematic review of diverse evidence will provide a structured framework to guide the implementation of pharmaceutical solutions across healthcare settings, ensuring that interventions are both evidence-based, contextually relevant and avoid a siloed perspective.

A strength of this protocol is its emphasis on integrating multiple perspectives, including those of researchers/clinicians and individuals with lived experience, through a collaboration with a patient association. This participatory approach aims to ensure relevance of the reasoning in practice, and that the refined programme theory remains grounded in real-world needs and priorities. Moreover, by combining a systematic review methodology with a realist analysis, this study goes beyond assessing intervention effectiveness to reveal the mechanisms underlying pharmaceutical solutions and their impact on mental health care pathways. This dual methodological feature enhances the potential applicability of findings to diverse healthcare contexts.

However, this study may face several anticipated challenges. First, the generalisability of the refined programme theory may be limited if the contexts of the CMO configurations are not sufficiently defined or are too specific to a particular health service setting. The concept of transferability will therefore need to be critically examined to determine how the findings might be adapted to other contexts. Second, the heterogeneity of pharmaceutical solutions and the diversity of their related study designs in the literature may be challenging to synthesise. To address this, the quality appraisal step must be conducted carefully, along with an iterative approach to refining CMOs in order to ensure robust and meaningful emerging patterns. Third, as the review relies exclusively on published material, it may be constrained by the known limitations of current service provision and reporting in the literature. Accordingly, our findings will not be interpreted as definitive measures of effectiveness, but rather as theory-driven explanations intended to inform future empirical research and service development.

Ultimately, given the lack of an existing comprehensive synthesis on pharmaceutical solutions and mental health care pathways, insights gained from this review could help prioritise pragmatic pharmaceutical interventions for implementation in different healthcare settings. These findings could inform the design of an integrated care model where relevant pharmaceutical interventions are systematically embedded within mental health care pathways.

### Ethics and dissemination

As this realist review will collect previously published data and will not involve human or animal participants, no ethical approval is required. Since this manuscript is a review protocol, no datasets were generated or analysed. All data extraction forms will be made available as part of the publication of the realist review.

## Supplementary material

10.1136/bmjopen-2025-110428 online supplemental table 1

10.1136/bmjopen-2025-110428 online supplemental file 1
