# Supplementary material for: Pharmaceutical solutions implemented to improve mental health care pathways: a systematic realist review protocol
Source: BMJ Open. 2025 Sep 30;15(9):e110428. doi: 10.1136/bmjopen-2025-110428 (PMC12496084; doi:10.1136/bmjopen-2025-110428)
Supplement: online supplemental file 1 [file bmjopen-15-9-s002.docx]

**Supplementary File 2:** Structured search terms used in PubMed, Web Of Science and Embase for step 3 (systematic review for evidence)

- Search terms used in **PubMed**

| ("Pharmaceutical Services"[MeSH] OR "Pharmacy and Therapeutics Committee"[Mesh] OR “pharmaceutical service*”[TIAB] OR “pharmaceutical solution*”[TIAB] OR “pharmaceutical act*”[TIAB] OR “pharmacist*”[TIAB] OR "Pharmacists"[MeSH] OR "Pharmacies"[TIAB] OR "Evidence-Based Pharmacy Practice"[Mesh] OR “pharmaceutical care"[TIAB] OR "pharmaceutical counseling"[TIAB] OR “pharmaceutical intervention*”[TIAB] OR "pharmacist-led intervention*"[TIAB] OR “pharmacistled”[TIAB] OR “pharmacist-driven”[TIAB] OR "Pharmaceutical follow-up"[TIAB] OR "Medication review"[TIAB] OR “Medication reconciliation*”[TIAB] OR "Pharma* workshop*"[TIAB] OR "Medication adherence support"[TIAB] OR “pharma* consultation*”[TIAB] OR “pharma* interview*”[TIAB] OR “pharma* intervention*”[TIAB] OR “pharma* assessment*”[TIAB] OR “clinical pharmacy”[TIAB] OR “hospital pharmac*”[TIAB] OR “community pharmac*”[TIAB] OR “pharmaceutical follow-up”[TIAB])  AND  (“mental dis*”[TIAB] OR "Mental Health"[Mesh] OR “mental health”[TIAB] OR "Addiction Medicine"[Mesh] OR “addict*”[TIAB] OR "Psychiatry"[Mesh] OR "psychiatr*"[TIAB] OR "Psychiatric Rehabilitation"[Mesh] OR "Mental Health Recovery"[Mesh] OR "Community Mental Health Centers"[Mesh] OR "Community Mental Health Services"[Mesh] OR "Mental Health Services"[Mesh] OR “mood disorder*”[TIAB] OR “bipolar”[TIAB] OR “depressive”[TIAB] OR “depression”[TIAB] OR “anxiety disorder*”[TIAB] OR “eating disorder*”[TIAB] OR “neurodevelopmental disorder*”[TIAB] OR “paraphilic”[TIAB] OR “personality disorder*”[TIAB] OR “schizophreni*”[TIAB] OR “substance use”[TIAB] OR “substance-related disorder*”[TIAB] OR “substance abuse”[TIAB] NOT “dementia”[TIAB])  AND  ("Patient Care Planning"[Mesh] OR “care pathway*”[TIAB] OR “clinical pathway*”[TIAB] OR “patient pathway*”[TIAB] OR “healthcare pathway*”[TIAB] OR “healthcare trajectory*”[TIAB] OR "Patient Care Management"[MeSH] OR "continuum of care"[TIAB] OR "care coordination*"[TIAB] OR “care management*”[TIAB] OR "patient journe*"[TIAB] OR “health journey”[TIAB] OR "Transitional Care"[Mesh] OR “transitional care”[TIAB] OR “care transition*”[TIAB] OR “coordination*”[TIAB] OR “care model*”[TIAB] OR “referral*”[TIAB] OR “management pathway*”[TIAB] OR “service delivery”[TIAB] OR “pathway* model*”[TIAB] OR “admission*”[TIAB] OR “readmission*”[TIAB] OR “hospitaliz*”[TIAB] OR “rehospitaliz*”[TIAB] OR “discharge*”[TIAB] OR “patient* transfer*”[TIAB] OR “pathway optimization*”[TIAB] OR "referr*"[TIAB])  AND  ("2015"[Date - Publication] : "2025"[Date - Publication]) |
| --- |

- Search terms used in **Web Of Science**

| TS=(“pharmaceutical service*” OR “pharmaceutical solution*” OR “pharmaceutical act*” OR “pharmacist*” OR "Evidence-Based Pharmacy Practice" OR "Pharmacy Research" OR “pharmaceutical care" OR "pharmaceutical counseling" OR “pharmaceutical intervention*” OR "pharmacist-led intervention*" OR “pharmacistled” OR “pharmacist-driven” OR "pharmaceutical follow-up" OR "Medication review" OR “Medication reconciliation*” OR "Pharma* workshop*" OR "Medication adherence support" OR “pharma* consultation*” OR “pharma* interview*” OR “medication counseling” OR “pharma* assessment*” OR “clinical pharmacy” OR “hospital pharmac*” OR “community pharmac*” OR “pharmaceutical follow-up”)  AND  TS=(“mental dis*” OR “mental health” OR “neurodevelopmental dis*” OR “addict*” OR "psychiatr*" OR "Psychiatric Rehabilitation" OR "Mental Health Recovery" OR "Community Mental Health Centers" OR "Community Mental Health Services" OR "Mental Health Services" OR “eating disorder*” OR “mood disorder*” OR “bipolar” OR “depressive” OR “depression” OR “paraphilic” OR “personality disorder*” OR “schizophreni*” OR “anxiety disorder*” OR “neurodevelopmental disorder*” OR “substance use” OR “substance-related disorder*” OR “substance abuse” NOT ‘dementia”)  AND  TS=("Patient Care Planning" OR “care pathway*” OR “clinical pathway*” OR “patient pathway*” OR “healthcare pathway*” OR “healthcare trajectory*” OR "Patient Care Management" OR "continuum of care" OR "care coordination*" OR “care management*” OR "patient journe*" OR “health journey” OR “transitional care” OR “care transition*” OR “coordination*” OR “care model*” OR “referral*” OR “management pathway*” OR “service delivery” OR “pathway* model*” OR “admission*” OR “readmission*” OR “hospitaliz*” OR “rehospitaliz*” OR “discharge*” OR “patient* transfer*” OR “pathway optimization*” OR “referr*”)  **FILTER** : “last 10 years” |
| --- |

- Search terms used in **Embase**

| ('pharmacy (shop)'/exp OR 'pharmacy and therapeutics committee'/exp OR 'pharmaceutical service*':ti,ab,kw OR 'pharmaceutical solution*':ti,ab,kw OR 'pharmaceutical act*':ti,ab,kw OR 'pharmacist*':ti,ab,kw OR 'pharmacist'/exp OR 'pharmacies':ti,ab,kw OR 'evidence-based pharmacy'/exp OR 'pharmaceutical care':ti,ab,kw OR 'pharmaceutical counseling':ti,ab,kw OR 'pharmaceutical intervention*':ti,ab,kw OR 'pharmacist-led intervention*':ti,ab,kw OR 'pharmacistled':ti,ab,kw OR 'pharmacist-driven':ti,ab,kw OR 'pharmaceutical follow-up':ti,ab,kw OR 'medication review':ti,ab,kw OR 'medication reconciliation*':ti,ab,kw OR 'pharma* workshop*':ti,ab,kw OR 'medication adherence support':ti,ab,kw OR 'pharma* consultation*':ti,ab,kw OR 'pharma* interview*':ti,ab,kw OR 'pharma* intervention*':ti,ab,kw OR 'pharma* assessment*':ti,ab,kw OR 'clinical pharmacy':ti,ab,kw OR 'hospital pharmac*':ti,ab,kw OR 'community pharmac*':ti,ab,kw OR 'pharmaceutical follow-up':ti,ab,kw)  AND  ('mental dis*':ti,ab,kw OR 'mental health'/exp OR 'mental health':ti,ab,kw OR 'neurodevelopmental dis*':ti,ab,kw OR 'addiction medicine'/exp OR 'addict*':ti,ab,kw OR 'psychiatry'/exp OR 'psychiatr*':ti,ab,kw OR 'psychosocial rehabilitation'/exp OR 'mental health recovery'/exp OR 'community mental health center'/exp OR 'community mental health service'/exp OR 'mental health service'/exp OR 'eating disorder*':ti,ab,kw OR 'mood disorder*':ti,ab,kw OR 'bipolar':ti,ab,kw OR 'depressive':ti,ab,kw OR 'depression':ti,ab,kw OR 'anxiety disorder*':ti,ab,kw OR 'paraphilic':ti,ab,kw OR 'personality disorder*':ti,ab,kw OR 'schizophreni*':ti,ab,kw OR 'substance use':ti,ab,kw OR 'substance-related disorder*':ti,ab,kw OR 'substance abuse':ti,ab,kw NOT 'dementia':ti,ab,kw)  AND  ('patient care planning'/exp OR 'care pathway*':ti,ab,kw OR 'clinical pathway*':ti,ab,kw OR 'patient pathway*':ti,ab,kw OR 'healthcare pathway*':ti,ab,kw OR 'healthcare trajectory*':ti,ab,kw OR 'patient care'/exp OR 'continuum of care':ti,ab,kw OR 'care coordination*':ti,ab,kw OR 'care management*':ti,ab,kw OR 'patient journe*':ti,ab,kw OR 'health journey':ti,ab,kw OR 'transitional care'/exp OR 'transitional care':ti,ab,kw OR 'care transition*':ti,ab,kw OR 'integrated health care system'/exp OR 'coordination*':ti,ab,kw OR 'care model*':ti,ab,kw OR 'referral*':ti,ab,kw OR 'management pathway*':ti,ab,kw OR 'service delivery':ti,ab,kw OR 'pathway* model*':ti,ab,kw OR 'admission*':ti,ab,kw OR 'readmission*':ti,ab,kw OR 'hospitaliz*':ti,ab,kw OR 'rehospitaliz*':ti,ab,kw OR 'discharge*':ti,ab,kw OR 'patient* transfer*':ti,ab,kw OR 'pathway optimization*':ti,ab,kw OR 'referr*':ti,ab,kw)  **FILTER** : “last 10 years” |
| --- |
