# Supplementary material for: Pharmaceutical solutions implemented to improve mental health care pathways: a systematic realist review protocol
Source: BMJ Open. 2025 Sep 30;15(9):e110428. doi: 10.1136/bmjopen-2025-110428 (PMC12496084; doi:10.1136/bmjopen-2025-110428)
Supplement: Supplementary file 3 [file bmjopen-2025-110428.reviewer_comments.pdf]

PEER REVIEW HISTORY

BMJ Open publishes all reviews undertaken for accepted manuscripts. Reviewers are asked to complete a checklist review form and are provided with free text boxes to elaborate on their assessment. These free text comments are reproduced below.

ARTICLE DETAILS

Title (Provisional)

Pharmaceutical solutions implemented to improve mental health care pathways: A systematic realist review protocol

Authors

LEBRAT, MATTHIEU; Carrouel, Florence; Franck , Nicolas; Dussart, Claude

---

VERSION 1 - REVIEW

**Reviewer**	**1**
**Name**	**Jean Spinks**
**Affiliation**	**The University of Queensland**
**Date**	**14-Aug-2025**
**COI**	**None**

Overall, I found the focus of this manuscript very narrow and ill-defined. Although the manuscript goes to great lengths to detail how integrated services can be incorporated into the review, the focus is only on "pharmaceutical care". The focus appears to be in pharmacists as providers of care, but "pharmaceutical care" is not clearly defined in the manuscript (certainly, some of the descriptors used seem to be country-specific and not readily understood more broadly). This specific focus on pharmaceutical care is not well justified. There is little discussion about the potential role(s) of pharmacists in mental health care and how that relates to their current or future scope of practice. For example, pharmacists do not typically diagnose mental heath conditions, nor are they typically trained to provide ongoing treatment (other than provision of medication and medication advice). So, the reader is left wondering if the focus is on how pharmaceutical care fits into more system-wide approaches or as stand-along services. It does not appear to be the former as the search strategy is limited to "pharmaceutical care". Such a siloed approach to health system evaluation and design does not help to solve the health system pressures currently being experienced due to the requirement for care for mental health conditions.

Further, if the authors are interested to help design better mental health care systems, it is not clear to me that a realist review offers the best methodology to address this aim. For example, a systems-thinking approach may be better suited to designing optimal care pathways. Relying on already published material as the source of all evidence given the known limitations of current service provision is illogical. I am also not convinced that genuine consumer involvement can be achieved through the realist review process due to the narrow focus on pharmaceutical care.

Overall, I did not assess the manuscript to contributing to the literature in this space and it is likely of little interest to readers of the BMJ Open.

**Reviewer**	**2**
**Name**	**Janet Long**

Affiliation **Australian Institute of Health Innovation, Australian Institute of Health Innovation**

Date **14-Aug-2025**

COI **None**

This is an interesting and well written protocol that has a rigorous design. References are good adn the methodological follows that recommended by RAMESES II. The purpose of each step is well understood and mostly, clearly articulated.
The main issue I have, which I believe is easily fixed with a few more sentences, is a lack of clear rationale and background information. The way it is currently stated (line 138ff) the focus is all on pharmaceutical solutions (which sounds like "I have a tool. What can I use it on?") rather than outcomes of improved health for people with mental health conditions. Reconsider the wording and maybe add to the rationale section.
Second, there is missing information / assumed knowledge about the background. What are the usual care pathways for a person with a mental health condition? Are you talking about acute inpatient treatments or residential rehab centres or community-based services or all of them? I understand this is part of the aim – to work out the context in which pharmaceutical solutions work but I think a high level view of the problem of mental health pathways needs to be given first. Maybe consider a graphic showing a patient moving through the Mental health system and the places where pharmaceutical interventions could be useful. See comments below. The setting needs to be very clearly spelled out and linked to the rationale of the study. It also requires a sentence speaking to the feasibility of the research knowing that a vague or unbounded research setting will land you in the realist swamp.
Line 103 Paragraph doesn't explain in broad terms what mental health pathways/ models of care might look like. BMJOpen has a general readership and so readers may not be aware of how acute or chronic mental health conditions are treated. Rehab is mentioned in the Abstract but not here. It would be useful to flesh out common pathways a person with a mental health condition might take and how pharmacy interventions can support that.
Line 115 "Pharmacists are key actors in healthcare systems." Can you add a sentence here clarifying that pharmacists work in a range of contexts – in hospitals, publicly funded community clinics and privately run commercial pharmacies so their access to patients and resources differ.
Not sure what you mean by "through their territorial network". Are you referring to their presence in community high streets (their "territories") or do you mean their expertise in medications and human responses as being territory distinct from medical?
This paragraph starting on Line 115, are these pharmacy-led initiatives, i.e., developed by pharmacists themselves? There isn't much detail about each one you give as an example – I realise it is part of the review to tease these things out, but I found it hard to really understand what the interventions were. Could you add a brief explanation of each (a phrase or short sentence). I think readers would be aware of medication reviews but not the other ones mentioned (i.e., discharge workshops, outpatient pharmaceutical followups).
Line 237. "C (Contexts), in which situations / conditions / environments are pharmaceutical solutions effective and trigger underlying mechanisms..." Check grammar.
Line 244 "discussions with the expert researchers and their laboratories…" The word laboratories implies they are bench scientists? Maybe need to rephase for an international audience to be clear who they are - whether they are pharmacists doing basic science research or clinical or what?
Line 299 "First, ML will screen the documents based on titles and abstracts, and if deemed suitable for our study, also based on full-text reading against the inclusion and exclusion criteria." Please amend the grammar here / reword to be clear.
Inclusion criteria include articles in French. Can you provide a brief comment on which of your team is fluent in both English and French? Maybe you all are? Will French language articles be treated differently? Will data be extracted in English to facilitate synthesis?
Line 326 Quality Appraisal. The addition of richness as an additional criterion is excellent and the reference helpful. Could you add in here a sentence explaining what features contribute to "richness"?
Line 433 Discussion. I found this limitation curious: "First, the generalizability of the refined program theory may be limited by disparities in healthcare systems. The concept of transferability will thus have to be critically examined to determine how findings can be adapted to different contexts." I suggest rewriting this in slightly different terms. The whole point of realist research is that in a tightly defined context you can be confident of the intended outcome. Maybe change the wording to say generalisability may not be possible is contexts of the CMOs are not sufficiently defined or are too specific to a particular health service

configuration.
Best wishes for your future research

VERSION 1 - AUTHOR RESPONSE

Point by point response to Reviewer 1:

Regarding the comment 1: "**Overall, I found the focus of this manuscript very narrow and ill-defined. Although the manuscript goes to great lengths to detail how integrated services can be incorporated into the review, the focus is only on "pharmaceutical care"**."

Author's response: The focus of the manuscript is intentionally narrow to have a precise study subject, namely a well-delimited component of the healthcare system: pharmacist- led interventions. We have provided further explanations to highlight why the focus is limited to "pharmaceutical care" and why we chose this specific component of healthcare systems in order to apply a rigorous methodology in our study. This can be found on pages 6-7, lines 123-147.

"Pharmacists are among the most accessible healthcare providers and are key stakeholders in healthcare systems, ensuring access to care through their extensive community-based network [16]. They work across a range of contexts, including hospitals and privately run commercial / community pharmacies, where their access to patients and resources may differ, influencing the scope of care they can provide. With an average of 58 to 123 pharmacists per 100,000 inhabitants across most European countries in 2022 [17], they contribute to prevention, follow-up, and treatment, making pharmaceutical solutions particularly valuable in mental health care pathways [18]. Pharmacists frequently encounter individuals with mental health concerns, sometimes even prior to diagnosis. For example, community pharmacists have reported seeing individuals with symptoms suggestive of anxiety or depression before a formal diagnosis [19].
Specifically, pharmacists provide pharmaceutical care, originally defined in the 1990s as "the direct, responsible provision of medication-related care for the purpose of achieving definite outcomes" [20]. Over time, the concept has broadened to encompass a wider range of pharmacist-led services that improve multiple health outcomes, not solely those related to drug therapy [21,22]. Thus, pharmaceutical care represents a shift from merely dispensing medications to proactively managing therapy within a patient-centered approach aimed at optimizing health outcomes. Pharmaceutical professionals themselves have been rethinking their role within care pathways, and the literature supports evidence of pharmacists' desire to better bridge the multiple unmet needs in mental health care [18,23,24].
The scope of this work has therefore been limited to studying the contribution of pharmaceutical care through specifically designed solutions intended to enhance mental health care pathways."

Regarding the comment 2: "**The focus appears to be in pharmacists as providers of care, but "pharmaceutical care" is not clearly defined in the manuscript (certainly, some of the descriptors used seem to be country-specific and not readily understood more broadly). This specific focus on pharmaceutical care is not well justified.**"

Author's response: We have added the definition of "pharmaceutical care" on page 7, lines 135–147, and provided a justification for focusing on the pharmaceutical component of healthcare systems when examining healthcare pathways in mental health.

“Specifically, pharmacists provide pharmaceutical care, originally defined in the 1990s as “the direct, responsible provision of medication-related care for the purpose of achieving definite outcomes” [20]. Over time, the concept has broadened to encompass a wider range of pharmacist-led services that improve multiple health outcomes, not solely those related to drug therapy [21,22]. Thus, pharmaceutical care represents a shift from merely dispensing medications to proactively managing therapy within a patient-centered approach aimed at optimizing health outcomes. Pharmaceutical professionals themselves have been rethinking their role within care pathways, and the literature supports evidence of pharmacists’ desire to better bridge the multiple unmet needs in mental health care [18,23,24].
The scope of this work has therefore been limited to studying the contribution of pharmaceutical care through specifically designed solutions intended to enhance mental health care pathways.”

Regarding the comment 3: “**There is little discussion about the potential role(s) of pharmacists in mental health care and how that relates to their current or future scope of practice. For example, pharmacists do not typically diagnose mental heath conditions, nor are they typically trained to provide ongoing treatment (other than provision of medication and medication advice). So, the reader is left wondering if the focus is on how pharmaceutical care fits into more system-wide approaches or as stand-along services. It does not appear to be the former as the search strategy is limited to “pharmaceutical care”. Such a siloed approach to health system evaluation and design does not help to solve the health system pressures currently being experienced due to the requirement for care for mental health conditions**.”

Author’s response: We agree that the potential roles of pharmacists in mental health care and how these align with their current and future scope of practice require further clarification. In response, we have revised the discussion section, and these revisions can be found on page 7, lines 139–144:

“Thus, pharmaceutical care represents a shift from merely dispensing medications to proactively managing therapy within a patient-centered approach aimed at optimizing health outcomes. Pharmaceutical professionals themselves have been rethinking their role within care pathways, and the literature supports evidence of pharmacists’ desire to better bridge the multiple unmet needs in mental health care [18,23,24].”

We have also adapted the discussion to better situate pharmaceutical care within broader system-wide approaches to mental health. While our focus remains on pharmaceutical interventions studied in a specific context, which is precisely the strength of a realist methodology, we now highlight both the complementary role of pharmacists and the importance of avoiding a siloed perspective. These revisions can be found on page 22, lines 475-483:

“In order to support this change, data must be obtained on (i) potential new interventions that pharmacists can take part in to enhance mental health care, (ii) how these activities can be successfully integrated within a broader, system-wide approach, and (iii) which outcomes should be used to evaluate their impact on care pathways.
The realist systematic review of diverse evidence will provide a structured framework to guide the implementation of pharmaceutical solutions across healthcare settings, ensuring that interventions are both evidence-based, contextually relevant and avoid a siloed perspective.”

Regarding the comment 4: “**Further, if the authors are interested to help design better**

mental health care systems, it is not clear to me that a realist review offers the best methodology to address this aim. For example, a systems-thinking approach may be better suited to designing optimal care pathways."

Author's response: We acknowledge that the rationale for employing a realist review may lack clarity in our protocol. We have therefore revised the *Study design* section accordingly (pages 9-10, lines 193–210):

"Pharmaceutical solutions are inherently complex, as they often involve multiple subprocesses and engage a range of stakeholders [14,25,27,31]. Moreover, they are delivered by humans, each bringing unique approaches and variations in care delivery, which adds further complexity to their implementation and effectiveness [32]. To develop a clear understanding of how pharmaceutical solutions work, and how they can be most effectively implemented within a system-wide approach, it is necessary to assess the relationship between the context in which the interventions are applied, the mechanisms by which they work and the outcomes which are produced. A realist review is specifically designed for this purpose, providing a valuable tool for synthesizing heterogeneous and multi-component evidence to analyze complex pharmaceutical solutions across diverse contexts [30,33]. Although systems-thinking approaches are relevant for higher-level policy design, they do not provide the same level of explanatory detail regarding why an intervention works for some groups and not others. The realist logic of inquiry has been successfully applied in mental health research (e.g., Howe et al., BMJ Qual Saf 2023 [13]) and is therefore appropriate for the present study. Importantly, the insights generated from this realist review could subsequently inform and complement systems-thinking approaches, ensuring that both methodological perspectives contribute to a more integrated understanding of mental health care pathways."

Furthermore, the realist approach is a validated methodology that is regularly used in health system evaluation and design to address system pressures in mental health. For example, the study by Howe et al. (BMJ Qual Saf. 2023;34(1):40–52), which we have included in our reference list, presents a realist review on medication optimisation for people living with severe mental illness in the community, exploring what works, for whom, and in what circumstances to support effective medication use.

Regarding the comment 5: "**Relying on already published material as the source of all evidence given the known limitations of current service provision is illogical.**"

Author's response: We agree that relying exclusively on published material presents limitations, particularly given the well-documented gaps in current service provision. However, the realist review methodology is specifically designed to synthesize heterogeneous evidence and to explore how and why interventions work (or fail to work) in different contexts (we highlighted this, pages 9-10, lines 200-203). By analyzing published evidence, we are able to identify context–mechanism–outcome configurations that provide valuable insights into the role of pharmaceutical care in mental health. We have now revised the protocol to make these limitations more explicit and to emphasize that our findings will not be interpreted as definitive statements of effectiveness, but rather as theory-driven explanations that can inform future empirical research and service development (see page 23, lines 502–506).

"Third, as the review relies exclusively on published material, it may be constrained by the known limitations of current service provision and reporting in the literature. Accordingly, our findings will not be interpreted as definitive measures of effectiveness, but rather as theory-driven explanations intended to inform future empirical research and service development."

Regarding the comment 6: "**I am also not convinced that genuine consumer involvement can be achieved through the realist review process due to the narrow focus on pharmaceutical care.**"

Author's response: We agree that consumer involvement within a realist review focusing on pharmaceutical solutions may be more limited than in broader health service evaluations. However, we believe consumer perspectives remain essential, particularly in understanding experiences with all the pharmaceutical aspects they experience during their care, such as medication use, perceptions of pharmacists' roles, and barriers to accessing care. Having inputs from individuals with lived experience is also an ethical consideration required by the journal.

In our protocol, consumer input is therefore incorporated not only to refine the initial program theory but also to help interpret emerging context–mechanism–outcome configurations. We have revised the manuscript to clarify the scope of consumer involvement (see page 20, lines 443–447):

"Patient involvement is embedded throughout the protocol via collaboration with a patient association, ensuring that the perspectives of individuals with lived experience inform the study. Their priorities, experiences, and preferences will guide the development and refinement of the program theory, with particular attention to shaping the emerging context–mechanism–outcome configurations."

Point by point response to Reviewer 2:

Regarding the comment 1: "**The main issue I have, which I believe is easily fixed with a few more sentences, is a lack of clear rationale and background information. The way it is currently stated (line 138ff) the focus is all on pharmaceutical solutions (which sounds like "I have a tool. What can I use it on?") rather than outcomes of improved health for people with mental health conditions. Reconsider the wording and maybe add to the rationale section.**"

Author's response: We acknowledge that in the current version, the rationale may overemphasize pharmaceutical solutions rather than explicitly linking them to the overarching aim of improving health outcomes for people with mental health conditions. In response, we have revised the rationale section in the introduction, as well as the wording of the objectives, (originally line 138ff) to better articulate how pharmaceutical solutions contributes to system-wide strategies for mental health, and to foreground the intended outcomes for patients. These changes ensure that the focus is placed on improving mental health outcomes, with pharmaceutical solutions framed as one component of a broader, outcome-driven approach (see pages 8-9, lines 178–187):

"This approach will support a clearer understanding of how mental health outcomes can be improved by optimizing care pathways, while also providing a structure to interpret our review findings. Within this broader objective, we will focus on the role of pharmaceutical solutions as one component of system-wide strategies in mental health. Accordingly, a systematic review will be conducted to identify pharmaceutical solutions that have been implemented to strengthen care pathways. The primary objective of this study is to determine how such pharmaceutical solutions contribute to improving outcomes for people with mental health conditions, specifically, what works, for whom, and in what context. The secondary objective will be to identify the key outcomes currently used to evaluate the impact of pharmaceutical solutions on care pathways."

Regarding the comment 2: "**Second, there is missing information / assumed knowledge about the background. What are the usual care pathways for a person with a mental health condition? Are you talking about acute inpatient treatments or residential rehab centres or community-based services or all of them? I understand this is part of the aim – to work out the context in which pharmaceutical solutions work but I think a high level view of the problem of mental health pathways needs to be given first. Maybe consider a graphic showing a patient moving through the Mental health system and the places where pharmaceutical interventions could be useful.**"

Author's response: We agree that additional background on mental health care pathways is needed to contextualize our discussion of pharmaceutical interventions. In response, we have:

1. Expanded the background section to provide a concise overview of typical care pathways for people with mental health conditions, including acute inpatient care, standard inpatient care, and outpatient care with community-based services (see pages 5-10, lines 104–113):

"People with mental health conditions typically move through a range of care pathways that vary in intensity and duration depending on clinical need. After diagnosis, usually by primary care providers, patients may be referred to one or more of the following settings: acute inpatient care for severe episodes (such as emergency department), standard longer-term inpatient care for a global therapeutic strategy decision or adaptation, and outpatient or community-based services for ongoing management and rehabilitation [8–10]. Patients may move between these settings to achieve therapeutic goals, with long-term follow-up often provided by multidisciplinary teams in primary or community care, focusing on patient-centered rehabilitation and relapse prevention."

2. Added a new figure (Figure 1) illustrating a simplified pathway through the mental health system, highlighting where pharmaceutical solutions may play a role alongside the different care settings:

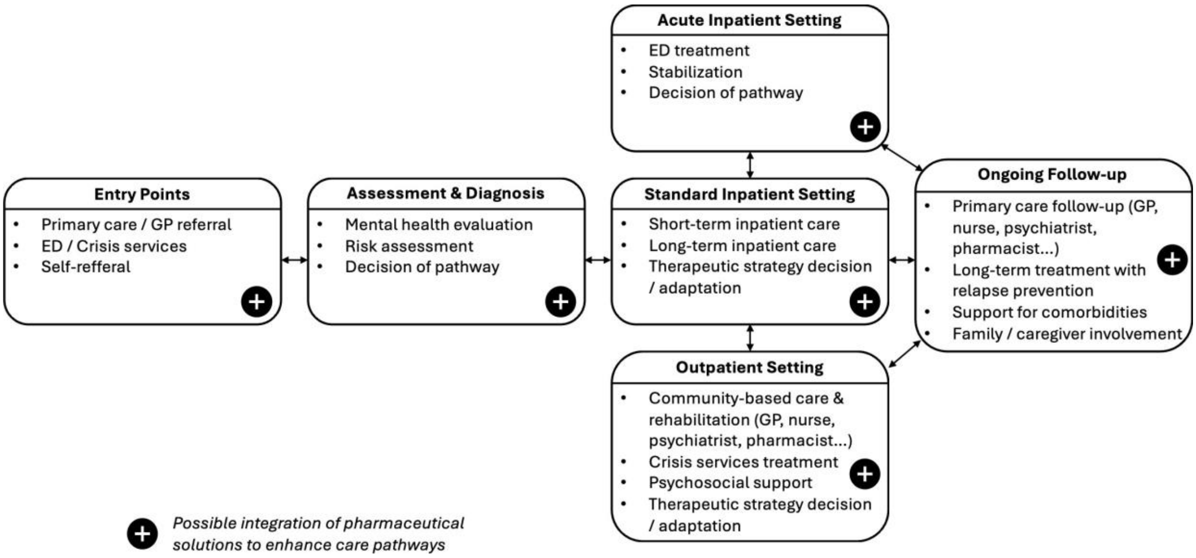

Figure 1. Simplified mental health care pathway showing entry points, care settings, and transitions.

We believe these additions improve the clarity and accessibility of the manuscript, particularly for readers less familiar with mental health care structures.

Regarding the comment 3: "**The setting needs to be very clearly spelled out and linked to the rationale of the study. It also requires a sentence speaking to the feasibility of the research knowing that a vague or unbounded research setting will land you in the realist swamp.**"

Author's response: The setting has been clarified in the updated introduction (Page 7, lines 145-147) and further detailed in the *study design* section of the Materials and Methods (Page 9, lines 193-200). To address feasibility, an additional sentence has been added in the *search strategy* section (Page 15, lines 325–327):

"Considering the combinations of our search terms, this review will cover the full mental health care pathway, but analysis will be structured around pharmaceutical solutions within these pathways. This approach will ensure a comprehensive yet bounded scope."

Regarding the comment 4: "**Line 103 Paragraph doesn't explain in broad terms what mental health pathways/ models of care might look like. BMJOpen has a general readership and so readers may not be aware of how acute or chronic mental health conditions are treated. Rehab is mentioned in the Abstract but not here. It would be useful to flesh out common pathways a person with a mental health condition might take and how pharmacy interventions can support that.**"

Author's response: We acknowledge the need for a broader explanation of mental health care pathways and models of care. Accordingly, we have added a new paragraph (Pages 5-6, lines 104–113) immediately following the section indicated:

"People with mental health conditions typically move through a range of care pathways that vary in intensity and duration depending on clinical need. After diagnosis, usually by primary care providers, patients may be referred to one or more of the following settings: acute inpatient care for severe episodes (such as emergency department), standard longer-term inpatient care for a global therapeutic strategy decision or adaptation, and outpatient or community-based services for ongoing management and rehabilitation [8–10]. Patients may move between these settings to achieve therapeutic goals, with long-term follow-up often provided by multidisciplinary teams in primary or community care, focusing on patient-centered rehabilitation and relapse prevention."

We have also emphasized the rehabilitation component, which is essential in mental health, in this paragraph (Page 6, lines 110 and 113) and again later (Page 6, line 119).

Finally, we have added a new Figure 1 (response to comment 2), which illustrates common mental health care pathways and demonstrates how pharmacy interventions can support patients across different stages of care.

Regarding the comment 5: "**Line 115 "Pharmacists are key actors in healthcare systems." Can you add a sentence here clarifying that pharmacists work in a range of contexts – in hospitals, publicly funded community clinics and privately run commercial pharmacies so their access to patients and resources differ.**"

Author's response: We have revised the paragraph accordingly to clarify that pharmacists

work across different contexts, which affects their access to patients and resources. The following sentence has been added after the first sentence of the paragraph (Page 6, lines 125–127):

“They work across a range of contexts, including hospitals and privately run commercial / community pharmacies, where their access to patients and resources may differ, influencing the scope of care they can provide.”

Regarding the comment 6: “**Not sure what you mean by “through their territorial network”. Are you referring to their presence in community high streets (their “territories”) or do you mean their expertise in medications and human responses as being territory distinct from medical?**”

Author’s response: We thank the reviewer for pointing out the ambiguity. We have revised the wording to clarify our intended meaning, now referring to pharmacists’ extensive community-based network (Page 6, lines 123–125):

“Pharmacists are among the most accessible healthcare providers and are key stakeholders in healthcare systems, ensuring access to care through their extensive community-based network [16].”

Regarding the comment 7: “**This paragraph starting on Line 115, are these pharmacy-led initiatives, i.e., developed by pharmacists themselves? There isn’t much detail about each one you give as an example – I realise it is part of the review to tease these things out, but I found it hard to really understand what the interventions were. Could you add a brief explanation of each (a phrase or short sentence). I think readers would be aware of medication reviews but not the other ones mentioned (i.e., discharge workshops, outpatient pharmaceutical followups).**”

Author’s response: We have clarified that these are pharmacist-led initiatives and have expanded the descriptions to briefly explain each intervention (Pages 7-8, lines 150–156). The revised text now reads:

“These solutions, developed by pharmacists, can include: targeted pharmaceutical consultations (pharmacist-led meetings with patients on a specific issue, e.g., antidepressant initiation), discharge workshops (inpatient workshops led by pharmacists in which patients preparing for discharge receive both individualized and general advice on their medications to support continuity of care), and outpatient pharmaceutical follow- ups (specific follow-up by community pharmacists at each medication refill).”

Regarding the comment 8: “**Line 237. “C (Contexts), in which situations / conditions / environments are pharmaceutical solutions effective and trigger underlying mechanisms...” Check grammar.**”

Author’s response: We have revised the sentence for grammatical clarity (Page 13, lines 288-293). The revised version now reads:

“After this first selection, CMO relationships will be established and further refined: C (Contexts), the situations, conditions, or environments in which pharmaceutical solutions are effective and trigger underlying mechanisms; M (Mechanisms), how pharmaceutical solutions work and link C to O; O (Outcomes), the different patterns of outcomes on care pathways associated with pharmaceutical solutions.”

Regarding the comment 9: "**Line 244 "discussions with the expert researchers and their laboratories…" The word laboratories implies they are bench scientists?**

Maybe need to rephase for an international audience to be clear who they are - whether they are pharmacists doing basic science research or clinical or what?"

<u>Author's response</u>: To avoid any ambiguity, we have clarified the wording. In the manuscript, "laboratories" referred to accredited research units affiliated with universities. We have therefore revised the text to read (Page 14, lines 296–298):

"As in step 1, discussions with the expert researchers and their affiliated academic research unit will also be undertaken to gather their feedback."

Regarding the comment 10: "**Line 299 "First, ML will screen the documents based on titles and abstracts, and if deemed suitable for our study, also based on full-text reading against the inclusion and exclusion criteria." Please amend the grammar here / reword to be clear.**"

<u>Author's response</u>: We have reworded the sentence for clarity (Page 16, lines 353–355):

"First, ML will screen the documents based on titles and abstracts, and, if considered relevant, will then review the full text against the inclusion and exclusion criteria."

Regarding the comment 11: "**Inclusion criteria include articles in French. Can you provide a brief comment on which of your team is fluent in both English and French? Maybe you all are? Will French language articles be treated differently? Will data be extracted in English to facilitate synthesis?**"

<u>Author's response</u>: All members of our research team are fluent in both French and English. Therefore, French-language articles will be screened, extracted, and analyzed in the same manner as English-language articles. Data from French articles will be translated into English during extraction to ensure consistency and facilitate synthesis. We revised the *inclusion and exclusion criteria* section for better clarity (Page 16, lines 360–362):

"Documents in French-language will be screened, extracted, and analyzed in the same manner as English-language articles. Data from French articles will be translated into English during extraction to ensure consistency and facilitate synthesis."

Regarding the comment 12: "**Line 326 Quality Appraisal. The addition of richness as an additional criterion is excellent and the reference helpful. Could you add in here a sentence explaining what features contribute to "richness"?**"

<u>Author's response</u>: A sentence has been added to clarify the features contributing to the richness criterion (Page 18, lines 393–395):

"Richness will be considered high when the resource meaningfully contributes to theory development and/or testing [42]."

Regarding the comment 13: "**Line 433 Discussion. I found this limitation curious: "First, the generalizability of the refined program theory may be limited by**

disparities in healthcare systems. The concept of transferability will thus have to be critically examined to determine how findings can be adapted to different contexts." I suggest rewriting this in slightly different terms. The whole point of realist research is that in a tightly defined context you can be confident of the intended outcome. Maybe change the wording to say generalisability may not be possible is contexts of the CMOs are not sufficiently defined or are too specific to a particular health service configuration."

Author's response: We have revised the paragraph to align more closely with the realist research perspective, emphasizing the specificity of CMO configurations. The updated text now reads (Page 22, lines 494–498):

"First, the generalizability of the refined program theory may be limited if the contexts of the CMO configurations are not sufficiently defined or are too specific to a particular health service setting. The concept of transferability will therefore need to be critically examined to determine how the findings might be adapted to other contexts."

VERSION 2 - REVIEW

Reviewer 2

Name Long, Janet

Affiliation Australian Institute of Health Innovation, Australian Institute of Health Innovation

Date 12-Sep-2025

COI

I thank the authors for engaging with my comments and answering them thoughtfully. I believe it is a much stronger protocol now. Very happy to recommend acceptance.
